# Comparison of Force During the Endotracheal Intubation of Commercial Simulation Manikins

**DOI:** 10.7759/cureus.43808

**Published:** 2023-08-20

**Authors:** Kate E Hughes, Md Tariqul Islam, Benjamin Co, Merryl Lopido, Neil L McNinch, David Biffar, Vignesh Subbian, Young-Jun Son, Jarrod M Mosier

**Affiliations:** 1 Emergency Medicine, University of Arizona, Tucson, USA; 2 Industrial Engineering, Purdue University, West Lafayette, USA; 3 Health Sciences, University of Arizona, Tucson, USA; 4 Biostatistics, McNinch Biostats, LLC (Limited Liability Company), Kent, USA; 5 Systems and Industrial Engineering, University of Arizona, Tucson, USA

**Keywords:** experienced clinicians, cormack-lehane grade, video laryngoscopy (vl), intubation force, intubation simulation, simulation trainer

## Abstract

Background

Medical simulation allows clinicians to safely practice the procedural skill of endotracheal intubation. Applied force to oropharyngeal structures increases the risk of patient harm, and video laryngoscopy (VL) requires less force to obtain a glottic view. It is unknown how much force is required to obtain a glottic view using commercially available simulation manikins and if variability exists. This study compares laryngoscopy force for a modified Cormack-Lehane (CL) grade I view in both normal and difficult airway scenarios between three commercially available simulation manikins.

Methods

Experienced clinicians (≥2 years experience) were recruited to participate from critical care, emergency medicine, and anesthesia specialties. A C-MAC size 3 VL blade was equipped with five force resistor reading (FSR) sensors (four concave surfaces, one convex), measuring resistance (Ohms) in response to applied pressure (1-100 Newtons). The study occurred in a university simulation lab. Using a randomized sequence, 49 physicians performed intubations on three manikins (Laerdal SimMan 3GPlus, Gaumard Hal S3201, CAE Apollo) in normal and difficult airway scenarios. The outcomes were sensor mean pressure, peak force, and CL grade. Summary statistics were calculated. Generalized estimating equations (GEEs) conducted for both scenarios assessed changes in pressure measured in three manikins while accounting for correlated responses of individuals assigned in random order. Paired t-test assessed for the in-manikin difference between scenarios. STATA/BE v17 (R) was used for analysis; results interpreted at type I error alpha is 0.05.

Results

Participants included 49 experienced clinicians. Mean years’ experience was 4(±6.6); median prior intubations were 80 (IQR 50-400). Mean individual sensor pressure varied within scenarios depending on manikin (p<0.001). Higher mean forces were used in difficult scenarios (603.4±128.9, 611.1±101.4, 467.5±72.4 FSR) than normal (462.5±121.9, 596.0±90.5, 290.6±63.2 FSR) for each manikin (p<0.001). All manikins required more peak force in the difficult scenario (p<0.03). The highest mean forces (Laerdal, CAE, difficult scenario) were associated with the higher frequency of grade 2A views (p<0.001). The Gaumard manikin was rated most realistic in terms of force required to intubate.

Conclusion

Commercially available high-fidelity manikins had significant variability in laryngoscopy force in both normal and difficult airway scenarios. In difficult airway scenarios, significant variability existed in CL grade between manikin brands. Experienced clinicians rated Gaumard Hal as the most realistic force applied during endotracheal intubation.

## Introduction

Endotracheal intubation is a frequently performed life-saving medical procedure in the emergency department. Clinicians need to acquire and maintain intubation skills to confidently and safely intubate critically ill patients. However, it is difficult to learn and practice this skill safely during clinical practice only, given the high risk associated with the procedure [1-4]. Medical simulation offers the opportunity to fill this gap, with the benefit of high-risk procedural training in a safe environment. Several commercially available manikin trainers exist, varying from isolated intubation-only task trainers to comprehensive high-fidelity manikins.

Intubation is most often performed using a rigid oral laryngoscope to overcome the anatomic obstacles imposed by the tongue and hypoepiglottic ligament to expose the glottic inlet. Traditionally this required a direct laryngoscope (DL) to compress the upper airway soft tissues, but more recently video laryngoscopes offer indirect visualization with less compression of the soft tissues. Studies show that the force applied to oropharyngeal structures increases patient risk of harm [5-9]. Additionally, most clinical studies show that VL is associated with improved procedural outcomes and requires less force on the oropharyngeal structures [10-21].

A knowledge gap remains, however, in understanding how the forces required to obtain a glottic view to successfully intubate compare between the commercially available manikins, which could potentially have significant implications in task training. It is unknown whether commercially available high-fidelity manikins provide comparable intubation training experiences for learners. Specifically, it is unknown if the force required by the laryngoscope to successfully view the airway structures (i.e., obtain an adequate Cormack-Lehane (CL) view of the airway) is similar across various manikins. Conversely, it is also unknown whether the CL view is comparable between manikins when similar intubation forces are applied.

The goal of this study is to fill this knowledge gap by comparing the laryngoscopy force required to obtain a CL I grade of view in both normal and difficult airway scenarios between three commercially available simulation manikins. We hypothesize that high-fidelity manikins do not provide consistent training experiences for learners in that the force required to obtain an equivalent airway view varies significantly between manikins. Secondarily, we hypothesize that CL view varies depending on manikin despite intubation expertise. This article was previously presented as a meeting abstract at the 2023 SAEM Annual Meeting on May 19, 2023.

## Materials and methods

At an academic institution’s simulation lab, experienced clinicians (defined as ≥2 years of experience and greater than 75 clinical intubations) were eligible to participate and recruited via email for a convenience sample size of 50. Physicians with less than two years of experience were excluded. Anesthesia, emergency medicine, pulmonary critical care, and relevant subspecialties (e.g., emergency medicine critical care) were eligible for participation. Written informed consent was obtained, and the study was approved by the University’s institutional review board.

**Figure 1:**
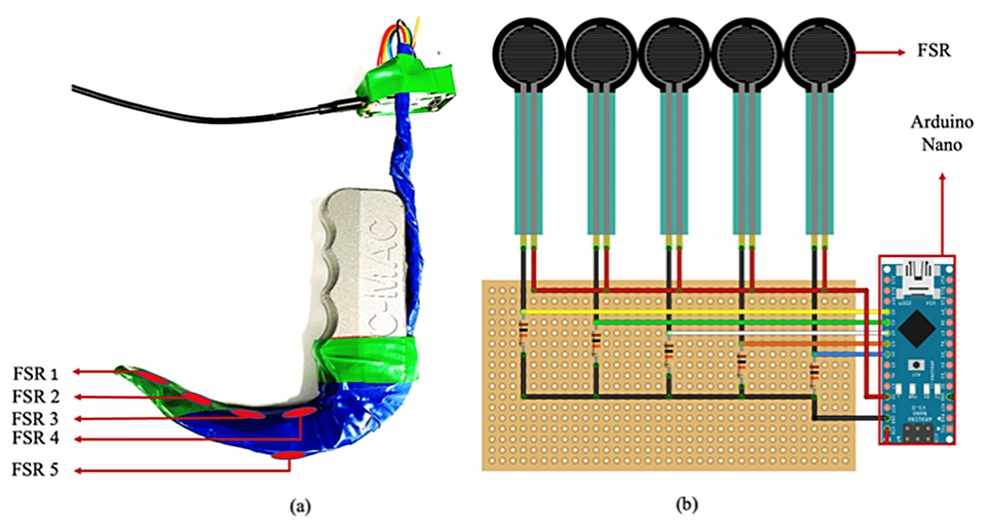
A. Modified C-MAC pressure sensing module B. Pressure sensing module circuit diagram FSR, force resistor reading

All participants used a C-MAC (Karl Storz, Tuttlingen, Germany) size 3 VL equipped with five resistance sensors (Interlink Electronics, Camarillo, CA, USA), four along the concave surface of the blade and one on the convex side for anterior teeth contact force measurement (Figure 1A). These resistors adjust their resistance value in response to the amount of pressure applied to them (measured in ohms) and can detect forces between 0-22 pounds (0-100 Newtons). The analog force resistor reading (FSR) is proportionate to the pressure applied, and values range from 0 to 1023. The pressure sensing module's circuit diagram is shown in Figure 1B. It includes an Arduino nano (Arduino, Sommerville, MA, USA), resistors, and five pressure sensors.

Microsoft Data Streamer, a two-way data transmission that streams live data from a microcontroller into Microsoft Excel (Microsoft Corporation, Redmond, USA), was used. Sensors were wired to a microcontroller connected to a Windows 10 personal computer to transfer data from the sensors into an Excel workbook. This enabled us to collect real-time data from the soft tissue deformation during the procedure, helping the parametrization of soft-tissue physics to capture realistic and accurate tissue movement. Three commercially available high-fidelity patient simulators were utilized for normal airway (tongue deflated) and difficult airway (tongue inflated) scenarios: CAE Apollo (CAE Healthcare Inc, Saint-Hubert, Quebec, Canada), Laerdal SimMan 3G Plus (Laerdal, Stavanger, Norway), Gaumard Hal S3201 (Gaumard Scientific, Miami, FL, USA).

All participants completed an online survey to collect demographic information and baseline data on prior experience and self-reported first-pass success prior to the experiments. Simple permutation randomization determined the order of laryngoscopy scenarios on each manikin and the order of manikin use. All participants were asked to obtain a modified CL grade I view, or their best view, of the glottic structures. Pressure profiles of all sensors were recorded during each intubation attempt until the point of the best-obtained view. Each participant was allotted a single attempt for each scenario, totaling six intubations. Each intubation was observed by a trained observer for CL modified grading of view obtained. Participants completed a post-experiment survey assessing each manikin’s feasibility for successful intubation and its realism of force to obtain the goal view compared to the clinical setting. Each participant was given a 50 USD gift card for participating in the study. The primary outcome of this study is defined as sensor pressure along the laryngoscope blade at the point of the goal view, with secondary outcomes of the best modified CL grade view obtained in each scenario, and rated manikin realism of the laryngoscopy force.

We calculated summary statistics (mean (sd) for continuous data; n (%) for categorical data) for demographic data. Generalized estimating equations (GEEs) were used to assess changes in force measured in three manikins (by sensors 1-5) while accounting for correlated responses of individuals assigned in random order to all three manikins. This analysis was conducted twice, for manikins with the normal airway scenario and again for the difficult airway scenario. A paired t-test was performed to compare the normal and difficult airway scenarios for each manikin individually. Analyses were conducted using STATA/BE v17 (R) with results interpreted at a type I error rate of alpha = 0.05 level of statistical significance. 

## Results

Forty-nine physicians participated in the study. Demographic data are summarized in Table 1, and self-rated intubation experience is in Table 2. Post-experiment survey summary statistics are in Table 3. The forces differed significantly between manikins in both the normal and difficult airway scenarios, both among individual sensors and the average force (Table 4). Table 5 shows the CL view did not vary significantly in the normal airway scenario for any manikin, however, CL views varied significantly in the difficult airway scenario (p<0.001). The distribution of CL grade views obtained varied (Table 5). All three manikins recorded significantly more peak force in the difficult airway scenario compared to the normal airway scenario (Table 6); however, the peak force did not vary significantly between manikins within the difficult airway scenario (Table 4). Sensors 2 and 3 recorded significantly different forces between airway scenarios for the Laerdal manikin; sensor 1 recorded significantly different forces between airway scenarios for the CAE manikin; sensor 2 recorded significantly different forces between airway scenarios for the Gaumard manikin. The Gaumard manikin was rated as the most realistic by experts with regard to the force required to intubate (Table 3).

**Table 1:** Participants' demographic data

Demographic data	N	%
Male	34	69.4
Female	15	30.6
Emergency medicine	31	63.3
Anesthesia	15	30.6
Pulmonary critical care	3	6.1
Attending physician	17	34.7
Fellow	5	10.2
Resident physician	27	55.1
Extremely uncomfortable performing intubations	3	6.1
Somewhat uncomfortable performing intubations	2	4.1
Neither comfortable nor uncomfortable performing intubations	3	6.1
Somewhat comfortable performing intubations	17	34.7
Extremely comfortable performing intubations	24	49
Somewhat familiar with simulation	9	18.8
Moderately familiar with simulation	28	58.3
Extremely familiar with simulation	11	22.9

**Table 2:** Participant self-rated intubation experience

	Mean	SD
Age	35.2	7.6
Years in clinical practice (excluding residency)	4	6.6
Number of clinical intubations	493	1472
Estimated first-pass success	89.9	12.6
Number of simulated intubations	35.9	51.5

**Table 3:** Manikin feasibility and realism Post-survey ratings by participants

	CAE Apollo	Laerdal SimMan 3G+	Gaumard Hal S3201
Highly unrealistic, N (%)	0	1 (2)	0
Somewhat unrealistic	8 (16.3)	5 (10.2)	6 (12.2)
Neither	4 (8.2	13 (26.5)	10 (20.4)
Somewhat realistic	35 (71.4)	27 (55.1)	28 (57.1)
Highly realistic	2 (4.1)	3 (6.1)	5 (10.2)
Most realistic regarding force required	13 (26.5)	10 (20.4)	24 (49)
Feasibility of intubation			
Strongly disagree	0	0	0
Somewhat disagree	5 (10.2)	3 (6.3)	1 (2.1)
Neither	3 (6.1)	3 (6.3)	3 (6.3)
Somewhat agree	12 (24.5)	16 (33.3)	22 (45.8)
Strongly agree	29 (59.2)	26 (54.2)	22 (45.8)

**Table 4:** Results of tests for differences in measured force using generalized estimating equations

Normal airway scenario (all manikins)	p-value	Difficult airway scenario (all manikins)	p-value
Sensor 1	0.361	Sensor 1	0.048
Sensor 2	< 0.001	Sensor 2	0.162
Sensor 3	< 0.001	Sensor 3	< 0.001
Sensor 4	< 0.001	Sensor 4	< 0.001
Sensor 5	0.004	Sensor 5	0.422
Average Force	<0.001	Average Force	<0.001
Peak Force	0.059	Peak Force	0.160

**Table 5:** Cl grade views by scenario CL, Cormack-Lehane

CL grade	CAE Apollo	Laerdal SimMan 3G+	Gaumard Hal S3201
Normal airway, N (%)			
I	27 (55.1)	26 (53.1)	36 (73.5)
IIa	16 (32.7)	22 (44.9)	9 (18.4)
IIb	4 (8.2)	1 (2.0)	2 (4.1)
III	2 (4.1)	0	2 (4.1)
IV	0	0	0
Difficult airway, N (%)			
I	10 (20.4)	12 (24.5)	35 (71.4)
IIa	27 (55.1)	28 (57.1)	11 (22.5)
IIb	9 (18.4)	6 (12.2)	1 (2.0)
III	3 (6.1)	1 (2.0)	2 (4.1)
IV	0	2 (4.1)	0

**Table 6:** Results of paired t-test to assess for differences in forces

Laerdal SimMan 3G Plus (Normal & difficult airway)	p-value	CAE Apollo (Normal & difficult airway)	p-value	Gaumard Hal (Normal & difficult airway)	p-value
Sensor 1	0.165	Sensor 1	<0.001	Sensor 1	0.110
Sensor 2	<0.001	Sensor 2	0.134	Sensor 2	<0.001
Sensor 3	<0.001	Sensor 3	0.475	Sensor 3	0.040
Sensor 4	0.107	Sensor 4	0.094	Sensor 4	0.726
Sensor 5	0.340	Sensor 5	0.403	Sensor 5	0.201
Average force	<0.001	Average force	0.345	Average force	<0.001
Peak force	0.001	Peak force	0.001	Peak force	0.026

## Discussion

Studies have not evaluated commercially available simulation manikins that learners commonly utilize for intubation simulation. While it has been previously published that VL causes a decreased force to oropharyngeal tissues compared to direct laryngoscopy, the literature has not reviewed whether the manikins that are used to teach this critical skill mirror clinical reality [20]. Using the most realistic training devices available for learners for a procedural skill is optimal to maintain fidelity in skill transition to clinical practice. We found that measured laryngoscopy forces do vary significantly between manikins in both normal and difficult airway scenarios. Perhaps these inconsistencies between high-fidelity manikins are due to variances in tissue composition and pliability with respect to deformation under laryngoscope blade pressure. We intentionally used experienced clinicians to avoid confounding by skill level [22]. Thus, the differences in force measurements likely represent a true difference between manikins rather than a difference in the skill of the participants. The implications of these findings are important for novices, who need realistic and reliable simulations to learn the procedural mechanics of intubation for optimal skill transfer to the clinical setting.

We found that the CL grade obtained varied significantly by manikin brand in the difficult airway setting specifically, despite the best attempts to obtain a CL I view. The Gaumard manikin had the least variability in CL grade view between normal and difficult scenarios (73.5% Grade 1 normal scenario to 71.4% Grade 1 difficult scenario). Overall, there was a statistically significant decrease in the number of Grade 1 views obtained for each brand of manikin between normal and difficult airway scenarios. This is consistent with previously published data that increased airway difficulty is associated with a poor laryngoscopy view [23]. Manikins maintain clinical realism in difficult airway settings (e.g., tongue swelling) to increase the technical difficulty of intubation, as evidenced by increased grade of modified CL view. This maintains clinical relevance, as first-pass success decreases and the incidence of hypoxia increases as the CL grade increases; manikins that have difficult airway settings associated with higher CL grades allow learners to practice difficult airway management techniques in a safe setting [23].

Each manikin recorded increased peak force in its difficult airway scenario compared to its normal airway scenario; however, the peak force did not vary significantly between brands of the manikin in the difficult airway scenario. It is sensible that increased peak force was used by participants when the manikin had difficult airway settings, to navigate tongue edema and attempt an improved CL grade view. In prior studies, the use of VL decreased peak force and tongue force during intubation although it was associated with higher maxillary incisor force [20,21]. The clinical significance of peak force is speculative; although the force is known to contribute to patient physiological stress response and oropharyngeal tissue trauma, the specific quantity remains to be determined. Russell et al. hypothesized different forces may cause different physiological effects but noted that reducing any force is likely beneficial for high-risk patients [20].

Participants in this study rated the Gaumard Hal S3201 manikin as the most realistic in terms of force required to intubate. All manikins were rated as feasible to perform the procedure successfully. Interestingly, a prior study reviewed the pharyngeal size of the Gaumard Hal manikin in comparison to actual patients using computed tomography measurements and found it to be significantly larger than that of the average patient, calling into question the fidelity of simulator-based procedural training [24]. However, our study focused solely on the force applied during intubation in comparing several manikins to each other and did not include patient-based data for force. Future studies should consider the inclusion of a clinical patient cohort using the C-MAC laryngoscope blade with sensors to assess for force differences between high-fidelity patient manikins and patients. This would allow for a comprehensive assessment of the fidelity of simulation manikins. Additionally, future studies may compare different VL devices as well or different-sized blades for each device. Such data would pave the way for personalizing dynamic manikin settings for the specific laryngoscope during simulations.

This study has several limitations. The FSR sensors utilized in this study measure pressure applied proportionately. However, they do not have a linear relationship as the sensor’s primary function is to differentiate between high- and low-pressure conditions rather than detecting applied force. This limits the generalizability of these data as many other studies report values in Newtons. Study generalizability is also limited due to single-center data. The survey data relied on self-reported ratings of first pass success and numbers of intubations; historically, self-reported data is subject to bias and is often underreported [25]. 

## Conclusions

In conclusion, three commercially available high-fidelity manikins had significant variability in laryngoscopy force in both normal and difficult airway scenarios when experts performed endotracheal intubation. In difficult airway scenarios, significant variability existed in CL grade between manikin brands. All manikins consistently required increased peak force in the difficult airway scenario and did not significantly differ from each other. Gaumard Hal S3201 was rated by expert clinicians as the most realistic manikin with regard to the force applied during endotracheal intubation. Future translational research studies may consider a cohort of clinical patients to assess for force differences between high-fidelity manikins and patients for objective fidelity assessment. 
